# Numerical Investigation of Effective Thermal Conductivity of Strut-Based Cellular Structures Designed by Spatial Voronoi Tessellation

**DOI:** 10.3390/ma14010138

**Published:** 2020-12-30

**Authors:** Minghao Zhang, Junteng Shang, Shiyue Guo, Boyoung Hur, Xuezheng Yue

**Affiliations:** 1 School of Materials Science and Engineering, University of Shanghai for Science and Technology, No. 516, Jungong Road, Shanghai 200082, China; lnzminghao@163.com (M.Z.); shangjunteng@outlook.com (J.S.); 2 Department of Aerospace Engineering, Tokyo Metropolitan University, Tokyo 191-0065, Japan; gsy_19910511@yahoo.co.jp; 3 Department of Metallurgical and Materials Engineering, Gyeongsang National University, Jinju 501, Korea; hurby@gnu.ac.kr

**Keywords:** cellular structure, Voronoi tessellation, heat transfer, effective thermal conductivity, finite element analysis

## Abstract

Porous materials possess light weight and excellent thermal insulation performance. For disordered porous structures, the number of seed points is an important design parameter which is closely related to the morphology and mean pore size of the structure. Based on the arrangement of points in three-dimensional space, seven kinds of structures were designed by spatial Voronoi tessellation in this paper. The effect of the number of seed points on effective thermal conductivity for Voronoi was studied. Numerical simulation was conducted to research the effects of structural porosity, filling material and structural orientation on the effective thermal conductivity and heat transfer characteristics. The results showed that the effective thermal conductivity is closely related to the porosity and the matrix material. Different number and arrangement of seed points make the structure have different anisotropic performance due to different thermal paths. In addition, required the least number of seed points was obtained for the designation of isotropic random Voronoi.

## 1. Introduction

Additive manufacturing (AM) constructs objects by stacking layers of materials [1,2]. AM has high design freedom in three-dimensional space [3]. Objects with complex internal structures can be easily designed by parameterized constructive solid geometry (CSG) [4] or computer tomography (CT) [5] and fabricated by AM. The fabrication of multiple parts with rapid integration is another significant advantage of AM compared with the traditional processing technologies such as investment casting [6], physical vapor deposition [7], and melt gas injection [8]. In terms of AM technologies, the ability to process multifarious materials has huge potential [9,10,11]. Porous materials possess the characteristics of optimized distributions of pore size, mutative pore morphology, relative density, and high specific surface [12], thus porous material display excellent acoustic [13], biomedical [14], thermal conductivity [15] and fluid transfer [16] properties. However, when considering the needs of porous materials in actual application scenarios, we need to find the characteristics of matching porous materials and optimize them. The utility of additive manufacturing technologies makes the precise control of the arbitrary characteristics of porous materials and fidelity structural processing possible.

The strut-based cellular structure is a porous material that has a self-supporting structure, with the advantage that it is not necessary to design a support structure [17], which simplifies the product development process and reduces the cost of materials. Strut-based cellular structures have attracted many researchers’ attention because their satisfactory thermal isolation applications and excellent mechanical properties. Bai et al. [18] analyzed several lattice structures and proposed a method capable of producing directional control thermal conductivity. Yun et al. [19] investigated the heat transfer and stress characteristics of the FCCZ lattice channel by varying the porosity and inlet velocity. Mirabolghasemi et.al. [20] proposed a mathematical model and compared the predictions of this model with the numerical homogenization which revealed that the cell pores alter the heat transfer. These studies have revealed that the pore size and relative density have a significant effect on the anisotropy on the effective thermal conductivity of porous materials.

The Voronoi tessellation technique is commonly applied to develop porous structure [21]. In general, due to the different algorithms the spatial Voronoi tessellations can be divided into Poisson-Voronoi tessellation (PVT) and Laguerre-Voronoi tessellation (LVT) [22]. Compared to the LVT, the space partitioned by PVT relies only on the number and locations of seed points, which is a simple process but the geometry obtained by PVT is different from the real foam structure in its morphological characteristics [22,23]. Nie et al. [24] investigated the pressure drop of open-cell foams created by LVT with various pore densities and porosities. Almonti et al. [25] proposed an indirect additive manufacturing method that combined the AM with metal casting to fabricate the open-cell foams designed by Voronoi tessellation and compared the resulting mechanical properties with commercial metal foams. These studies revealed that the porosity and pore size are significant structural parameters that are closely related to the property of the open-cell foam.

Titanium and its alloys are widely used in aerospace and orthopedic medicine, due to their excellent mechanical properties, light weight, biocompatibility and heat transfer properties [26]. Wang et al. [27] investigated triply periodic minimal surface (TPMS) which the selection of 30% porosity unit cells in a regenerative cooling structure was with Ti-6Al-4V. Karami et al. [28] studied selective laser melting of Ti-6Al-4V of diamond lattice structures and the effect of post-processing on microstructural anisotropy. However, there are few reports on the heat transfer performance of the cellular structure of the Kelvin and the three-dimensional (3D) Voronoi structure using titanium alloy materials. The application of additively manufactured complex titanium alloy components in the aerospace field is affected by complex stress conditions and thermal conditions. This study started from the point cloud design methods of three-dimensional Voronoi and Kelvin structure generation. Through researching the relationship between the variation in the distribution of struts and effective thermal conductivity, providing essential engineering application reference for design to structure heat transfer and stress characteristics. We migrate the methods of investigating the anisotropy of stress characteristics to the heat transfer, thereby the structure design of the seed points distribution utilizes this study considering the engineering application of both heat conduction and mechanics simultaneously.

Anisotropy refers to the physical, chemical, and mechanical properties of materials that will change direction [29,30,31]. The mechanical properties of materials are isotropic and can be applied to complex stress conditions, while materials with anisotropy need to consider the directionality of force loading in actual applications to avoid structural failure [32,33]. The thermal conductivity of the structure is related to the path of heat conduction, and its conduction path is also related to the distribution of the struts, so the heat conduction of the structure will also exist certain anisotropy problems [34]. The influence of porosity, pore volume distribution and direction on the effective thermal conductivity in open-cell porous materials were systematically studied by Skibinski et al. [35]. Venkataraman et al. [36] proposed an optimality criterion for minimizing heat conduction through the metal foam. Lu et al. [37] proposed the approximate closed-form solutions for the overall heat transfer coefficient and pressure drop as functions of cell morphologies in cellular structures. Many researchers have discussed the anisotropy of the mechanical properties of the strut-based cellular structures [32,38], so it is important to discuss the anisotropy of the strut-based cellular structures in the routes of heat conduction and ameliorate measures. This work will provide a positive reference for the application of strut-based structures with multi-physics of heat and force.

Previous researchers have studied the heat transfer characteristics of additive manufacturing (selective laser melting and selective laser sintering) strut-based cellular structures using experimental research and theoretical analysis methods, and mainly pay attention to their heat transfer characteristics [39,40,41]. The influence of the porosity, pore size and pore size distribution of 3D Voronoi structure on heat transfer has been extensively studied [24,25,35]. However, the research on the influence of the number of seed points on the heat transfer in the strut-based structure may be ignored. The number of seed points is an important design parameter in the Voronoi structure design process which determines the randomness of the structure. Therefore, fewer seed points may cause differences in the performance of the structure in different directions.

In this paper, the Kelvin and random Voronoi of strut-based structures models were designed by spatial Voronoi tessellation according to the arrangement of points in three-dimensional space. The distribution of the struts is obviously different in Kelvin and random Voronoi models. The dot matrix of these two kinds of strut-based structure uses orderly and random generation methods respectively. Each type of strut-based structure is designed with different thickness gradients and different rotation directions of the struts to change the distribution of struts in the space. Afterward, through the digital file of the 3D model, the two structures random Voronoi and Kelvin are numerically simulated for the steady-state thermal conduct. The influence of five kinds of different porosity, the filling material and orientation on the effective thermal conductivity, and heat transfer characteristics were analyzed and evaluated. Moreover, for random Voronoi structure, it also has researched the influence of number of seed points on the effective thermal conductivity.

## 2. Materials and Methods

### 2.1. Design of Cellular Structures

**Figure 1:**
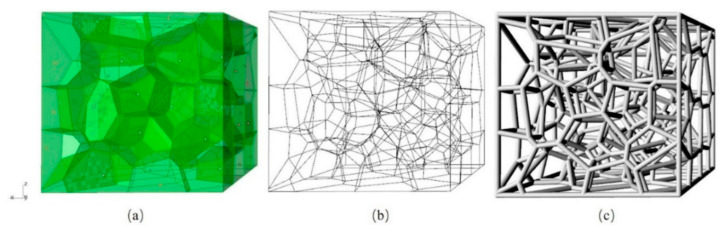
Design process of random Voronoi structure: (**a**) randomly distributed points and polyhedral cells; (**b**) frame lines of random Voronoi structure; (**c**) random Voronoi structure.

In this work, 3D Voronoi structures are chosen because they can be used to represent real cellular structures. We used different methods to generate two kinds of 3D Voronoi structures, namely, random and ordered Voronoi structures. The software Rhinoceros 5 (McNeal, Seattle, WA, USA) with plug-in Grasshopper (V 0.9.0076) was applied to design these structures. The Voronoi tessellation can be mathematically defined as [42,43]:

C_i_ = {P|d(P,S_i_) ≤ d(P,S_j_), ∀ j ≠ i, j = 1,…,n}, (1)

where, {S_i_,…,S_n_} is a set of seed points which are defined in 3D Euclidean space; d(P,S_i_) is the Euclidean distance between the location P and seed point S_i_; C_i_ is the Voronoi cell associated with seed point S_i_. The design process of random Voronoi strut-based cellular structures is illustrated in Figure 1. Firstly, a certain number of randomly distributed points were created in a space corresponding to the specimen. These points are called seed points in the Voronoi tessellation and a function of the number of pores per inch (PPI). Based on the locations of these points, whose numbers were equal to seed points. These polyhedral cells were controlled by seed points on the cell’s center. Subsequently, spatial lines of each polyhedron cell were extracted, overlapping lines were removed and the remaining lines were combined to obtain the frame lines of random Voronoi structure. After the above operation, a certain thickness was added to the frame lines to obtain a random Voronoi structure. However, the design method of spatial Voronoi tessellation can not only be applied to design the random structure but also a structure with a regular shape can be designed according to its principle of space division. For a designated space with the same length, width and height, the points are arranged at the eight vertices of the cube and the center of the cube, and space was divided into nine polyhedrons by spatial Voronoi tessellation through these points, and the polyhedron at the center of the body was extracted and a certain thickness was added to the frame lines to obtain the Kelvin structures.

**Figure 2:**
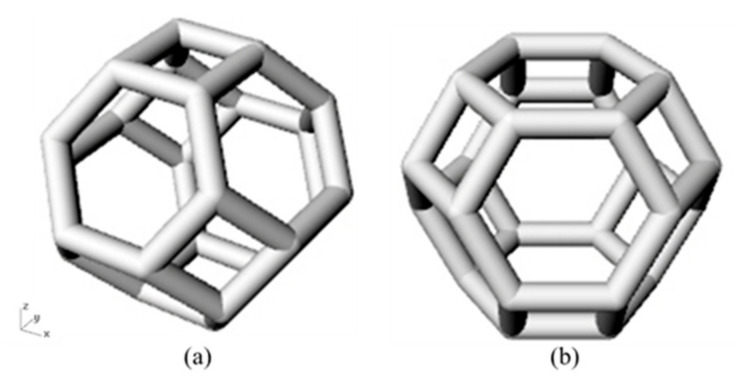
The unit cell of Kelvin structures: (**a**) Kelvin structure with tetragon as base; (**b**) Kelvin structure with hexagon as base.

Then the original Kelvin structure was rotated at a specific angle which is between the plane of the hexagonal side of the structure and the horizontal plane, as shown in Figure 2a,b, respectively. Then scale them to a suitable size so that they are arranged in the designated space in a 3 × 3 × 3 arrangement. The cross-sections of the above strut-based cellular structures were designed to be circular and the whole dimensions of all samples are 30 × 30 × 30 mm^3^.

**Figure 3:**
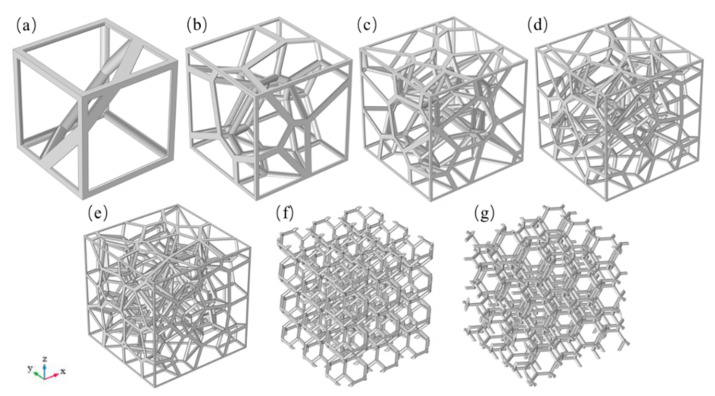
Strut-based cellular structures: (**a**) random Voronoi cellular structure with 2 seed points (RV2); (**b**) random Voronoi cellular structure with 10 seed points (RV10); (**c**) random Voronoi cellular structure with 30 seed points (RV30); (**d**) random Voronoi cellular structure with 50 seed points (RV50); (**e**) random Voronoi cellular structure with 70 seed points (RV70); (**f**) Kelvin structure with tetragon as base (KT); (**g**) Kelvin structure with hexagon as base (KH).

Computer-aided design (CAD) models of specimens prepared for numerical simulation are shown in Figure 3. All specimens are cubic, with a side length of 30 mm. The lightweight characteristics and performance of the cellular structure are closely related to its own porosity. Hence, in order to study the relationship between structural porosity and thermal behavior, fixing the frame line of the models and vary the radius of the struts allowed to design CAD models with a porosity ranging from 95% to 87%. The porosity of the cellular structure can be defined as:

P = (V_m_ − V_cs_)/V_m_ (2)

where V_m_ is the total volume of the space, V_cs_ is the volume of the cellular structure. In addition, the number of seed points is one of the important parameters for the design of the random Voronoi structure. We used the same algorithm to create 2, 10, 30, 50 and 70 seed points respectively to generate random Voronoi structures to study the effect of the number of seed points on the heat transfer in the random Voronoi structures. The specific parameters of the structures studied in this paper are shown in Table 1.

### 2.2. Heat Transfer Finite Element Modeling

**Figure 4:**
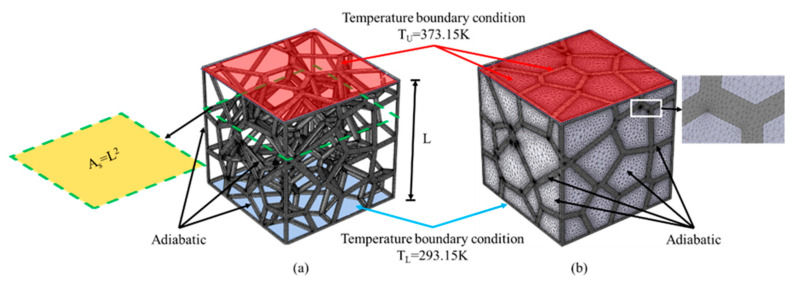
Boundary conditions and geometry parameter for RV50 structure in different environments: (**a**) Vacuum and (**b**) Air-saturated.

To investigate the effective thermal conductivity and steady-state heat transfer characteristics of the strut-based cellular structures under vacuum and air-saturated environments, the commercial software COMSOL^®^ v. 5.4 (COMSOL Inc., Stockholm, Sweden) was applied for numerical simulation. Figure 4 illustrates the boundary condition and geometry parameter of the RV50 structure under vacuum and air-saturated environments. The constant thermal loads of 375.15 K and 293.15 K were applied on the top and bottom surfaces of the models respectively, while other surfaces were set as adiabatic. Ti-6Al-4V was selected as the matrix material due to its high mechanical strength, high specific strength and low thermal conductivity [44]. The thermal conductivity, specific heat capacity and density of Ti-6Al-4V dependent on temperature are listed in Table 2.

**Figure 5:**
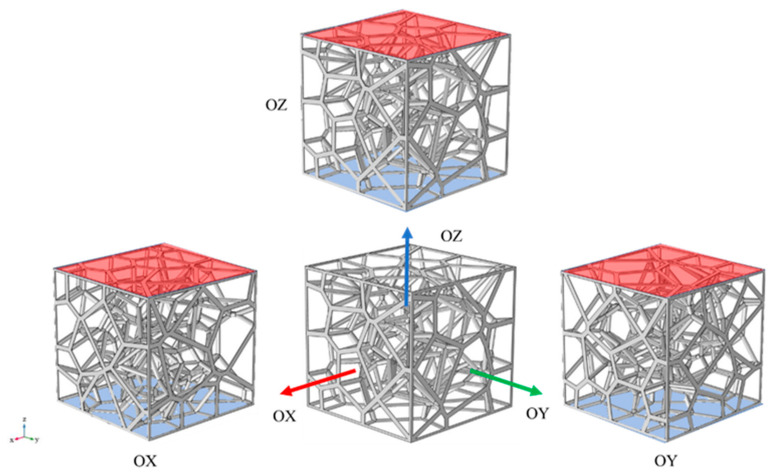
RV50 structure with a porosity of 94.82% in the OX, OY, and OZ orientation, the upper and lower surfaces of the models were applied to constant thermal loads of 375.15 K and 293.15 K respectively.

Generally speaking, due to the randomness of the structure, the physical properties of random structures do not have any orientation dependence. Out of interest in whether the random Voronoi structure shows significant differences in the thermal conductivity in different orientations, we have researched the effective thermal conductivities of the cellular structures of different types, porosity, and orientations. As shown in Figure 5, where we take the RV50 structure with a porosity of 94.98% as a representative substance, we specify that the orientation of all structures mentioned early is OZ, and the structure in the OZ orientation was rotated by 1.5 π around the y axis in the Cartesian coordinate system, after which it obtained the model of the corresponding structure in the OX direction. Similarly, the structure in the OZ orientation was rotated by 0.5 π around the X axis in the Cartesian coordinate system to generate the CAD model of the corresponding structure in the OY orientation. Figure 5 illustrates the CAD model and temperature boundary conditions of the RV50 structure with a porosity of 94.98% after being rotated, and the temperature boundary conditions are the same as those in Figure 4. What is worth pointing out is that referring to the two cellular structures in Figure 3c,d, their geometric models in the orientation of OX and OY are the same. For which, it only researches on their effective thermal conductivities and heat transfer characteristics in the OZ and OY orientation.

In this work, in order to simplify the calculation of the numerical simulation, we proposed the following assumptions: The effect of heat radiation inside the structure can be ignored considering that the temperature range was set at 293.15–373.15 K. In addition, since the direction of the temperature gradient is opposite to the direction of gravity, the airflow phenomenon caused by the change of air density could be ignored. In other words, the air is assumed to be stationary during the heat transfer process. Hence, ignoring the influence of thermal convection on heat transfer. According to the above assumptions, we only consider the heat conduction along the struts in the heat transfer process. Based on the energy conservation equation of steady-state heat conduction, the governing equation can be defined as [45]:

ρCp·▽T + ▽·(−k▽T) = Q(3)

where: ρ, C_p_, k is the density, specific heat capacity, and thermal conductivity of the material, respectively. ▽is the gradient operator, T is the temperature and Q is the heat generated by the heat source.

The effective thermal conductivity of different structures at a steady state can be calculated by Fourier’s law. When the heat conduction reaches equilibrium, the total heat flow Φ through the specimen can be given as:

(4) $$\Phi \;=\;-k_{\mathrm{eff}}\frac{\Delta T}{L}A_{s}$$

where: k_eff_ is the effective thermal conductivity of the structure, and ∆T is the temperature difference between the upper and lower surfaces of the model, namely, ∆T = T_U_ − T_L_; L is the height of the specimen, and A_s_ is the cross-sectional area of the space occupied by the specimen, namely A_s_ = 9 × 10^–4^ m^2^. In addition, according to the physical meaning of heat flow, Φ can also be expressed as:

(5) $$\Phi \;=\;\int_{A}\mathrm{qdA}$$

where: q is the heat flux of any section parallel to the upper and lower surfaces of the model. By substituting Equation (5) into Equation (4), the effective thermal conductivity can be expressed as:

(6) $$k_{\mathrm{eff}}\;=\;-\frac{\int_{A}q\cdot \mathrm{dA}}{\Delta T\cdot A_{s}}$$

### 2.3. Mesh Independence Tests

The mesh independence test was performed to evaluate the reliability of numerical simulation results. In general, as the element size decreases, the calculation time will dramatically increase and the calculation results will be more accurate. However, the accuracy of the calculation result will be in convergence with the decrease of the element size. When the element size decreases to the threshold, the calculation result will gradually become stable due to the decrease of the element size. Hence, in order to ensure the accuracy of the calculation results and save the calculation time, the mesh independence test is an indispensable part of our work.

**Figure 6:**
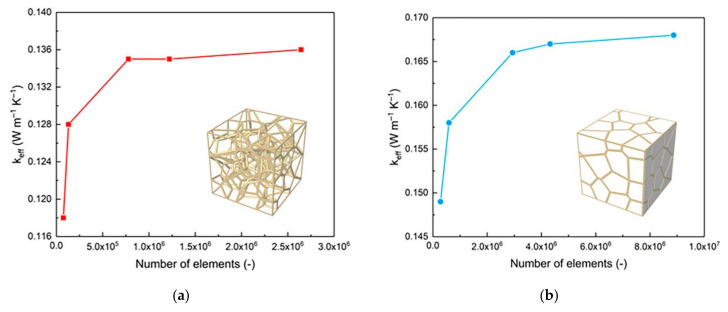
Mesh independence tests based on effective thermal conductivity: (**a**) Vacuum; (**b**) Air-saturated.

The tetrahedral element was chosen for finite element models because of its great adaptability. The effective thermal conductivity of the structure was employed as the indicator for judging whether the calculation results independent of the size of the element. Meshes with various sizes were selected for the RV50 structure with 94.98% porosity in both vacuum and air-saturated environment. In this work, five groups of mesh were used for mesh independence tests. For vacuum, the total number of elements are 76,118, 131,375, 778,750, 1,221,129, and 2,642,635, respectively. For air-saturated, the total number of elements are 281,189, 592,746, 2,937,363, 4,320,353, 8,871,323, respectively. Figure 6 shows the variation of the value of effective thermal conductivity with the different element sizes in both vacuum and air-saturated environment. The results show that when the number of elements less than 778,750 and 2,937,363 for vacuum and air-saturated environments, the deviations of the calculation results are 8.51% and 6.04%, respectively. When the number of elements is greater or equal to 778,750 and 2,937,363 for vacuum and air-saturated environment, the effective thermal conductivity becomes constant, and the maximum deviations of the calculation results are 0.74% and 1.2%, respectively. Hence, the meshes with 778,750 and 2,937,363 elements are considered as the optimal meshes which are used in the finite element molding.

## 3. Results

### 3.1. Effects of Filling Material on the Effective Thermal Conductivity

**Figure 7:**
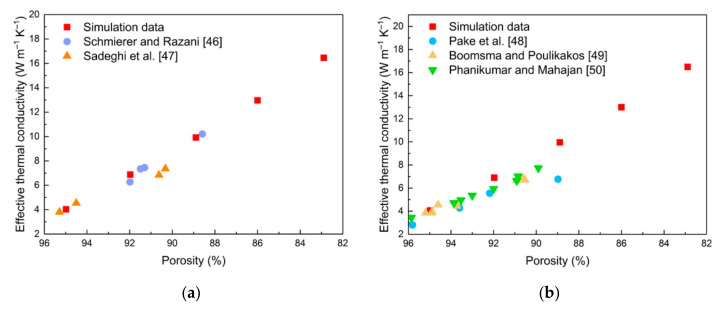
Comparison of simulation data and experimental data in the literature: (**a**) Vacuum; (**b**) Air-saturated.

At present, there are few studies on the thermal properties of the cellular structure of Ti-6Al-4V. To verify the accuracy of the simulation results and research the influence of air on the effective thermal conductivity, the physical properties of aluminum 6101 in literature [39] and the method described in Section 2.2 were used in finite element modeling. The RV50 structure was chosen as a representative to compare its effective thermal conductivity with existing experimental data from the literature [46,47,48,49,50], and the results are shown in Figure 7. The simulation data from this work show good agreement with experimental data in literature in a vacuum environment. Nevertheless, when considering the influence of air on heat conduction, the simulation data and the experimental data show slight differences and all of the errors are basically within 15%, which may be due to the simplification of the model and the ideal boundary conditions in the numerical simulation that ignore the thermal loss [39]. The simulation results showed that when the porosity of the RV50 structure decreases from 94.98% to 82.89%, the contribution of air to effective thermal conductivity decreases accordingly from 0.78% to 0.2%. Therefore, it can be concluded that when the structural porosity lower than 95% and the matrix material has high thermal conductivity such as aluminum 6101, the thermal conductivity of matrix material is far higher than that of air. Resulting in the contribution of air to effective thermal conductivity could be ignored.

For the matrix material composed of Ti-6Al-4V, the simulation results of the structures studied in this paper are shown in Table 3. Taking the RV70 structures in the OZ orientation as an example, the corresponding effective thermal conductivities of the RV70 structure with the porosity of 95.08%, 92.12%, 88.92%, 85.95% and 83.09% under vacuum and in an air-saturated environment are listed in Table 3. The presence of air improves the effective thermal conductivity of the structure by 22.96%, 13.60%, 9.52%, 7.2% and 5.96%, respectively. The calculation results show that referring to all structures, the difference of the effective thermal conductivities under the above-mentioned two environments is within 0.029–0.036 W m^−1^ K^−1^. Therefore, when metals with low thermal conductivity such as Ti-6Al-4V are used as the matrix material for cellular structures, and the cellular structures have relatively high porosity (>90%), the presence of air increases the effective thermal conductivity of the whole structure by more than 10%. Therefore, the contribution of air to effective thermal conductivity cannot be ignored.

### 3.2. Effect of the Number of Seed Points on the Effective Thermal Conductivity

**Figure 8:**
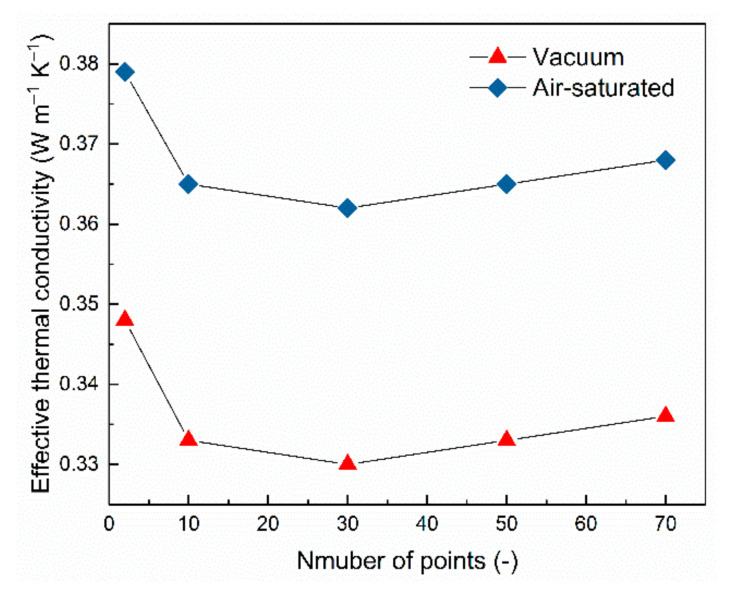
The effect of the number of seed points on the effective thermal conductivities of the RV structures.

The random Voronoi structures researched in this paper were generated based on randomly distributed points in space. Each point corresponds to a polyhedral cell. Hence, it takes the RV2, RV10, RV30, RV50 and RV70 structures with the same randomly point distributed pattern and different numbers of seed points as the research objects, to research the effect of the number of seed points on the effective thermal conductivity. The effective thermal conductivities of the random Voronoi structures with porosity of 89% in the OZ orientation under the vacuum and air-saturated environment are shown in Figure 8. The deviation between RV2 and RV30 under vacuum and the air-saturated environment is 5.45% and 4.50%, respectively. It is worth pointing out that the RV structure can only be generated when the number of seed points is greater than one. When the number of seed points was set as 2, the geometry of RV2 structure is similar to the cubic cell which makes it have the shortest thermal path in the above five structures. Hence, the effective thermal conductivity of RV2 structure is significantly higher than that of the other structures, for which the magnitude of the deviations corresponding to the vacuum and the air-saturated environment is only 1.82% and 1.66%, respectively, which appear in the RV30 and RV50 structures. In fact, the difference in the number of seed points causes a significant change in the geometric topology of the structure which alters the thermal path. On the other hand, when the porosity is fixed, the number of struts increases rapidly as the number of seed points increases, while the radius of the struts decreases. As a result, the overall thermal resistance of various RV structures are roughly the same that make similar effective thermal conductivities. Therefore, it concludes that the number of seed points has no obvious effect on the effective thermal conductivity of the structure when the number of points is greater than or equal to 10, that is, the pore density has no obvious relationship with the effective thermal conductivities of the random Voronoi structures, but this result is not inaccurate due to the anisotropic performance of effective thermal conductivity for RV10 and RV30 structures and it will be discussed in the next section. On the other hand, the increase in the number of seed points can strengthen the randomness of the structure, so that the structure has a better isotropic performance.

### 3.3. Effect of Porosity and Orientation on the Effective Thermal Conductivity

**Figure 9:**
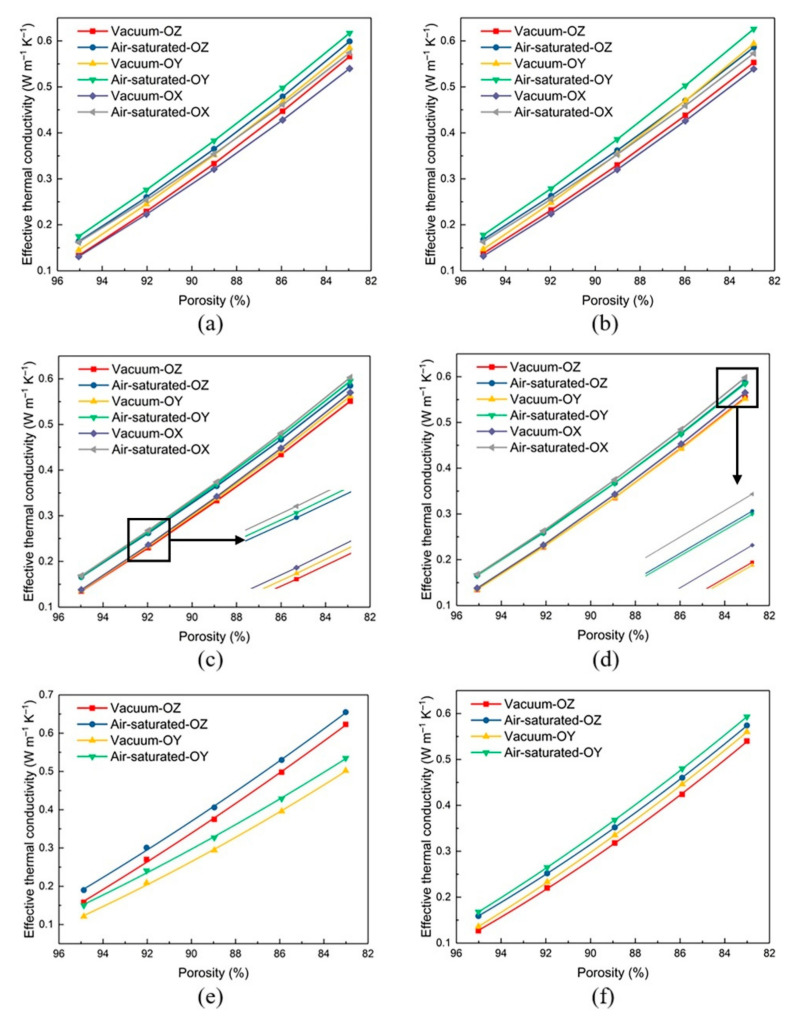
Effective thermal conductivity of various cellular structures in different orientations: (**a**) RV10; (**b**) RV30; (**c**) RV50; (**d**) RV70; (**e**) KT; (**f**) KH.

The corresponding effective thermal conductivities of different porosity, orientation, filling materials and structure types are shown in Figure 9. Taking the RV50 structure under the air-saturated environment as representation, as the porosity of the structure decreases from 94.98% to 82.89%, its effective thermal conductivity in the OZ orientation increases from 0.166 W m^−1^ K^−1^ to 0.585 W m^−1^ K^−1^ accordingly. The effective thermal conductivity of the structure is closely related to the volume fraction of the solid material, and thicker struts mean a higher fraction. As a result, the higher fraction of solid material lower fraction of air causes the greater effective thermal conductivity of the structure. In addition, it can be obviously observed in Figure 9b that the effective thermal conductivity of the RV30 structure in the OY orientation has a higher increase rate than other directions, which can be attributed to the RV30 structure having a shorter thermal transfer path in the OY direction [34].

The corresponding effective thermal conductivities of RV structures with various porosity in the OX, OY and OZ orientation were plotted in Figure 9a–d. We can find that both the RV structures showed great isotropic performance when their porosities are around 95%: under the air-saturated environment, the maximum differences of the effective thermal conductivity for four structures with 95% porosity in various orientations are 0.014 W m^−1^ K^−1^, 0.014 W m^−1^ K^−1^, 0.003 W m^−1^ K^−1^ and 0.002 W m^−1^ K^−1^, respectively; while for the vacuum environment, the maximum differences are 0.014 W m^−1^ K^−1^, 0.015 W m^−1^ K^−1^, 0.003 W m^−1^ K^−1^ and 0.003 W m^−1^ K^−1^, respectively. In addition, under the vacuum environment, the maximum deviations between RV10 and RV30 structures caused by the orientations are 10.69% and 11.36%, which are significantly higher than the RV50 and RV70 corresponding to 3.45% and 2.36%. Similar results can be observed in an air-saturated environment: the corresponding maximum deviations of RV10 and RV30 structures are 8.52% and 9.46%, and for RV50 and RV70 structures they are 3.08% and 2.22%. It’s worth pointing out that the differences in effective thermal conductivity of RV10 and RV30 structures caused by orientation are both greater than 5%. Hence, it can be considered that those structures show anisotropic effective thermal conductivity performance. These results reveal that more seed points impart stronger randomness for the structure due to the random distribution of the struts, resulting in a weakening of the influence of directions on the effective thermal conductivity. Furthermore, from Figure 9 we can find that as the porosity decreases, the effective thermal conductivity values in different directions start to “diverge” which may be attributed to the existence of various thermal transfer paths for each structure. In this work, the porosity will increase with the radius of the strut, for different heat transfer paths, the increase of strut radius contributes differently to the heat conduction. Hence, the effective thermal conductivities show different distribution trends in different directions changes with the porosity. 

Figure 9e,f illustrate the effective thermal conductivities of the KT and KH structures in the OZ and OY orientations when the porosity at the range of 95–83%. These results indicate that there exists a significant difference in the effective thermal conductivity values of the KT and KH structures in the OZ and OY orientations. When the porosity gradually decreases, the deviation will be gradually increased for the effective thermal conductivity in different orientations. It is worth noting that although the differences raise higher between the effective thermal conductivity values gradually, the orientations of the structure show a weaker influence on the effective thermal conductivity with the porosity decreasing accordingly. For the KT structure, the effective thermal conductivity in the OZ orientation is significantly higher than that in the OY orientation. Under the air-saturated environment, when the porosity is 94.87%, 92.03%, 88.96%, 85.91% and 83.01%, the ratio of the effective thermal conductivity in the two orientation are 26.67%, 24.90%, 24.16%, 23.54% and 22.43% respectively. For the KH structure, under the air-saturated environment, when the porosity is 95.01%, 91.94%, 88.92%, 85.9% and 83.01%, the corresponding effective thermal conductivity in the OZ orientation is 5.66% and 5.16%, 4.55%, 4.35% and 3.31% higher than that in the OY orientation respectively. These results showed that the KH structure appeared more stable thermal behavior than that of the KT structure while worse than the other random structures of RV50 and RV70 in the OY and OZ orientations when the porosity of the structure at the range of 95–83% in terms of the heat conduction. In addition, the KH and KT structures are the corresponding minimal effective thermal conductivity values than those of the other three structures in the OZ and OY orientations, respectively.

### 3.4. Temperature Distribution

**Figure 10:**
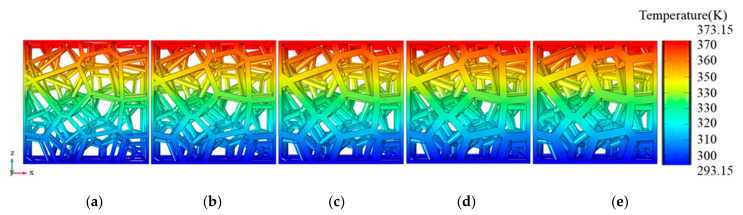
The temperature distribution of the RV50 structure with different porosity in air-saturation environment, the porosity values from left to right are (**a**) 94.98%, (**b**) 91.97%, (**c**) 88.89%, (**d**) 86% and (**e**) 82.89%.

**Figure 11:**
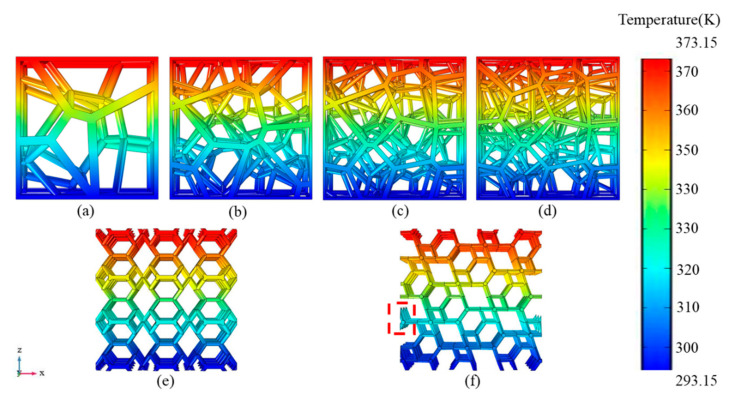
Temperature distribution of different structure types when the porosity is fixed at 95%: (**a**) RV10; (**b**) RV30; (**c**) RV50; (**d**) RV70; (**e**) KT; (**f**) KH.

In order to explore the effects of the model geometric parameters on heat transfer, the RV50 structure is taken as a representation under the air-saturated environment. The temperature distribution of RV50 structures with various porosity is shown in Figure 10. The thermal loads of constant temperature 373.15 K and 293.15 K were respectively applied to the top and the bottom surfaces. The temperature value decreases from the upper surface to the lower surface in an approximately linear distribution. Moreover, as the porosity decreases constantly, there is no significant difference in temperature distribution. The temperature distribution of different types of structures when the porosity is fixed at 95% as illustrated in Figure 11. The temperature distribution of the cellular structures with different structure types is almost the same, the temperature decreases from 375.15 K on the upper surface to 273.15 K in an approximately uniform distribution. Interestingly, the temperature distribution of the KH structure is slightly different from that of the other five structures, which is caused by the local discontinuity of the KH structure shown in the dashed line of Figure 11. Under the vacuum environment, the temperature is constant at 315.82 K in this region; and when the structure is filled with air, the existence of the air differentiates the temperature of this region slightly.

## 4. Discussion

As an important parameter of cellular structures, the porosity is closely related to the performance of the cellular structure. Therefore, it is necessary to study the effects of porosity on structure performance. As has been mentioned in Section 2.1, the porosity of the strut-based cellular structure is controlled by changing the strut radius. Hence, when the porosity changes, the geometric parameters of the structure will alteration inevitably. Referring to the strut-based cellular structure under the vacuum environment, the heat is transferred from the high-temperature side to the low-temperature side along the struts. However, as the radius of the strut increases, its cross-sectional area changes accordingly. The thermal resistance R of each strut is obtained based on the thermal Ohm’s law can be expressed as:

(7) $$R\;=\;\frac{l}{k\cdot S}$$

where: l is the length of the strut, k is the thermal conductivity of the matrix material and S is the cross-sectional area of the strut. Therefore, as the radius of the strut increases, the thermal resistance decreases accordingly, resulting in the effective thermal conductivity of the structure is increases as the porosity decreases. However, in terms of the strut-based cellular structure under the air-saturated environment, the heat can not only be transferred along the strut through the structure but also can be transferred through the air. Similarly, the decrease of the structural porosity is controlled by increasing the radius of the strut, resulting in a decrease of the thermal resistance of the strut. On the other hand, the decreases of the porosity refer to the increases in the volume of the matrix material and the decreases in the volume of the air. The thermal conductivity of air as the filler material is greatly different from that of the matrix material. The effects of the change of the effective thermal conductivity caused due to the decreases of the air volume are not as large as the increase in the volume fraction of the matrix material. Therefore, the effective thermal conductivity of the structure still increases with the decreases of the structural porosity. Then, in order to obtain a stronger thermal insulation performance, we need to increase the porosity of the structure. It is worth noticing that though the radius of the strut will gradually decrease as the porosity increases the effective thermal conductivity of the structure will decrease accordingly. However, excessive porosity will make the struts of the structure too thin, which may result in poor mechanical properties.

When cellular structures have the same porosity, the thermal performance will be affected by the microstructure and orientation of the structure because heat conduction has different heat transfer paths in different structures and orientations, resulting in slight differences in the effective thermal conductivity.

**Figure 12:**
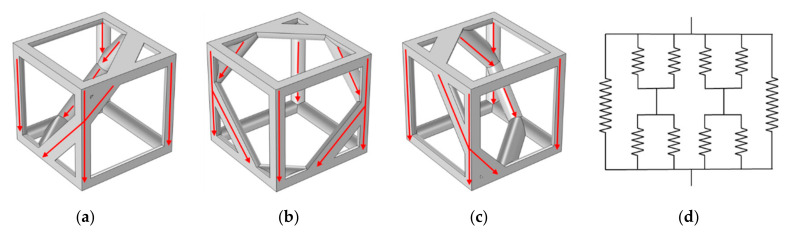
Schematic of the equivalent thermal cycle and the effects of different orientations of the RV2 structure on the heat transfer path: (**a**) OX; (**b**) OY; (**c**) OZ; (**d**) equivalent thermal circuit.

The RV2 structure with a porosity of 89% was taken as a representative to analyze the thermal resistances in OX, OY and OZ orientations to further illustrate the influence of orientations on the heat transfer path. Figure 12 illustrates the main heat transfer paths in different orientations for RV2 structure, although all struts can be equivalent to four groups of parallel in parallel during the heat transfer process in three directions, the two groups of thermal resistances can be further equivalent to two groups of thermal resistances in series. Nevertheless, the different structural orientations lead to changes in the distribution of each strut in space, and the combinations of thermal resistances for series and parallel were altered, which results in a slight difference in the effective thermal conductivity of the structure in different orientations.

The results in Section 3.4 show that among the RV50, RV70, KH and KT structures, the KT structure has the largest effective thermal conductivity in the OZ orientation, while the effective thermal conductivity in the OY orientation is the smallest. When the porosity in the range of 95–83%, the difference between the two values is more than 20%, which shows a strong anisotropic performance. Referring to the RV50 structure and the RV70 structure under the air-saturated environment, the largest difference of their effective thermal conductivities in different orientations is 3.89% and 2.22%, respectively, which shows a great isotropic performance. The reason for this difference may be due to different heat transfer paths in a different orientation. For the KT structure, its regular geometric topology makes it have very different thermal paths in the OZ and OY orientations, resulting in their overall thermal resistance being very different in each orientation. Similarly, for RV50 and RV70 structure, the randomness of the random Voronoi structures makes the overall thermal resistance in all orientations approximately equal, which results in tiny differences between the effective thermal conductivity in the OX, OY and OZ orientation of the random Voronoi structure. However, the difference in effective thermal conductivity of RV10 and RV30 structures caused by orientation are both greater than 5%, that those structures show anisotropic effective thermal conductivity performance. Therefore, the increase in the number of seed points makes the distribution of struts in the structure more random, the difference between the thermal resistance in different orientations is smaller, and the influence of the orientation of the structure on the effective thermal conductivity is gradually weakened. It is worth noting that the results in Section 3.3 show that the number of seed points has no significant effect on the effective thermal conductivity of the structure when the number of points is greater than or equal to 10. However, for anisotropic structures such as RV10 and RV30, it is meaningless to discuss the influence of the number of seed points on the effective thermal conductivity, because the orientation of the structure is also one of the main factors affecting its effective thermal conductivity. While existing studies have shown that the PPI is inseparable from the mechanical properties of the random Voronoi structure, especially the energy absorption capacity [25]. As has been mentioned above, the effective thermal conductivity of the structure is inversely proportional to the porosity. Referring to the random Voronoi structure, if the structure has better thermal insulation performance, it is necessary to further increase the porosity, resulting in the reduction of the strut radius. If by reducing the number of seed points, we reduce the number of struts in the random Voronoi structure, therefore the structure has thicker struts with the same porosity, which may improve the energy absorption and yield strength of the structure while not having a greater impact on the effective thermal conductivity of the structure. Nevertheless, fewer seed points may lead to a directional dependence on the performance of the structure. Therefore, how to balance the thermal insulation and mechanical properties of the random Voronoi structure should be considered more carefully.

In conclusion, usage of the lightweight characteristics of the strut-based cellular structure and the poor thermal conductivity of Ti-6Al-4V, and the design method based on spatial Voronoi tessellation to design the random Voronoi structure have been reported. It can be used in thermal protection systems, because of its lightweight and great thermal insulation performance. Moreover, by replacing the random distribution with the arrangement of the designated points in space, the structure with a specific heat transfer path can be customized to meet the expected thermal performance demands.

## 5. Conclusions

In this work, the method based on the spatial Voronoi tessellation was applied to design random Voronoi structures and ordered Voronoi structures. Numerical simulations based on the finite element method were conducted to study the effects of structural porosity, filling material and structural orientation on the effective thermal conductivity and heat transfer characteristics. Moreover, for seed Voronoi structures, the research of the effect of the number of seed points on the effective thermal conductivity has also been conducted. The main conclusions are as follows:

(1) As the porosity decreases, the effective thermal conductivity of the strut-based cellular structures increases. When it is within the high porosity range (>90%) and under the air-saturated environment, the effective thermal conductivity of the structure is more than 10% higher than that under the vacuum environment. At this time, we cannot neglect the contribution of air for heat conduction.
(2) Consider the influence of structural direction on effective thermal conductivity for the random Voronoi structure, when the number of seed points was set as 50 and 70, the difference between the effective thermal conductivity of the various structures is not obvious (<5%), the number of seed points of the structure has no obvious relationship with the effective thermal conductivity when the number of seed points are greater than or equal to 50.
(3) The thermal performance will be affected by the microstructure and orientation of the structure. Because of the heat conduction has different heat transfer paths in different structures and orientations, resulting in slight differences in the effective thermal conductivity. Referring to the RV50 and RV70 structures in an air-saturated environment, within the porosity range studied in this paper, the largest differences in their effective thermal conductivities in different orientations are 3.89% and 2.22%, respectively, showing good isotropic performance, and this performance is proportional to the number of seed points. The RV10 and RV30 structures show anisotropic effective thermal conductivity performance. Therefore, at least 50 seed points are needed to design a random Voronoi structure with isotropic properties. On the other hand, the effective thermal conductivities of KT and KH are more sensitive to the orientation, because the differences in effective thermal conductivities in different orientations are more obvious. The largest differences are 26.67% and 5.66%, respectively. The effective thermal conductivities of the KH and KT structures in the OZ and OY orientations are lower than that of the other five structures in the corresponding orientations.
(4) The porosity has no obvious effect on the temperature distribution. The temperature distribution trends are basically the same for the different structure types. The temperature value decreases from the top surface to the bottom surface in an approximately linear manner. However, in some inconsecutive areas on the KH structure, the temperature values are equal without any temperature gradient.

## Figures and Tables

**Table 1:** Specific geometrical parameters of the CAD models.

Structures	PPI	Porosity [%]	Strut Radius [mm]	Volume of the Cellular Structures [mm^3^]	Surface Area [mm^2^]
RV2 structure	1.07	95.00	1.79	1351.38	2958.81
		92.02	2.31	2154.97	3624.45
		88.97	2.77	2977.3	4140.52
		85.99	3.18	3781.43	4544.65
		82.97	3.57	4596.8	4886.34
RV10 structure	1.82	95.05	0.93	1335.26	4063.27
		92.03	1.20	2151.78	5021.67
		89.01	1.43	2966.35	5747.91
		85.95	1.64	3793.11	6352.50
		82.96	1.83	4601.27	6848.86
RV30 structure	2.63	94.99	0.62	1352.08	5356.45
		91.97	0.80	2169.25	6574.01
		89.02	0.95	2963.36	7474.76
		85.98	1.09	3785.41	8234.37
		82.93	1.22	4609.5	8868.72
RV50 structure	3.12	94.98	0.51	1355.63	6180.35
		91.97	0.66	2167.57	7558.77
		88.89	0.79	2999.46	8629.87
		86.00	0.90	3779.25	9443.53
		82.89	1.01	4619.81	10,173.77
RV70 structure	3.49	95.08	0.45	1329.73	6738.12
		92.12	0.58	2128.07	8247.22
		88.92	0.70	2992.49	9466.01
		85.95	0.80	3792.67	10,363.64
		83.09	0.89	4566.31	11,083.66
KT structure	-	94.87	0.41	1387.01	6678.86
		92.03	0.53	2237.68	8251.04
		88.96	0.62	2981.27	9319.81
		85.91	0.71	3805.43	10,294.41
		83.01	0.79	4587.59	10,984.59
KH structure	-	95.01	0.41	1346.38	6363.98
		91.94	0.53	2177.02	7872.74
		88.92	0.63	2990.45	9005.40
		85.90	0.72	3805.52	9930.22
		83.01	0.80	4585.99	10,676.37

**Table 2:** Temperature dependent physical properties of Ti-6Al-4V [45].

Temperature [K]	273.15	293.15	313.15	333.15	353.15	373.15	393.15
k [W m^−1^ K^−1^]	7.040	7.076	7.147	7.285	7.441	7.613	7.800
Cp [J kg^−1^ K^−1^]	525.117	536.041	545.973	553.497	560.723	567.660	574.316
ρ [kg m^−3^]	4432.103	4429.989	4428.525	4425.977	4423.419	4420.850	4418.271

**Table 3:** Effective thermal conductivity of cellular structures with various porosity, number of seed points and orientation.

Structures	PPI	Porosity [%]	Simulation Data of k_eff_ [W m^−1^ K^−1^] in Vacuum			Simulation Data of k_eff_ [W m^−1^ K^−1^] in Air-Saturated		
			OX	OY	OZ	OX	OY	OZ
RV2 structure	1.07	95.00	0.144	0.141	0.143	0.175	0.171	0.174
		92.02	0.240	0.238	0.241	0.271	0.269	0.268
		88.97	0.345	0.346	0.348	0.377	0.378	0.379
		85.99	0.454	0.461	0.459	0.485	0.493	0.491
		82.97	0.569	0.586	0.579	0.600	0.618	0.611
RV10 structure	1.82	95.05	0.131	0.145	0.133	0.162	0.175	0.164
		92.03	0.223	0.245	0.229	0.254	0.276	0.260
		89.01	0.321	0.352	0.333	0.354	0.383	0.365
		85.95	0.428	0.467	0.447	0.461	0.498	0.479
		82.96	0.540	0.585	0.566	0.574	0.617	0.599
RV30 structure	2.63	94.99	0.132	0.147	0.138	0.163	0.178	0.168
		91.97	0.224	0.248	0.232	0.256	0.279	0.263
		89.02	0.320	0.354	0.330	0.353	0.386	0.362
		85.98	0.426	0.470	0.438	0.459	0.503	0.470
		82.93	0.539	0.594	0.553	0.573	0.626	0.586
RV50 structure	3.12	94.98	0.138	0.136	0.135	0.169	0.167	0.166
		91.97	0.236	0.233	0.23	0.268	0.265	0.262
		88.89	0.342	0.338	0.333	0.374	0.370	0.365
		86.00	0.448	0.442	0.434	0.481	0.475	0.467
		82.89	0.570	0.562	0.551	0.603	0.595	0.585
RV70 structure	3.49	95.08	0.138	0.135	0.135	0.168	0.166	0.166
		92.12	0.232	0.228	0.228	0.263	0.260	0.259
		88.92	0.343	0.336	0.336	0.375	0.368	0.368
		85.95	0.453	0.443	0.444	0.485	0.475	0.476
		83.09	0.565	0.552	0.554	0.598	0.585	0.587
KT structure	-	94.87	-	0.121	0.158	-	0.150	0.19
		92.03	-	0.209	0.27	-	0.241	0.301
		88.96	-	0.294	0.375	-	0.327	0.406
		85.91	-	0.396	0.498	-	0.429	0.53
		83.01	-	0.502	0.623	-	0.535	0.655
KH structure	-	95.01	-	0.136	0.127	-	0.168	0.159
		91.94	-	0.233	0.22	-	0.265	0.252
		88.92	-	0.335	0.318	-	0.368	0.352
		85.90	-	0.446	0.424	-	0.480	0.460
		83.01	-	0.560	0.54	-	0.593	0.574

## Data Availability

Data sharing is not applicable to this article.
